# Sorafenib Neoadjuvant Therapy in the Treatment of High Risk Renal Cell Carcinoma

**DOI:** 10.1371/journal.pone.0115896

**Published:** 2015-02-03

**Authors:** Yushi Zhang, Yongqiang Li, Jianhua Deng, Zhigang Ji, Hongyan Yu, Hanzhong Li

**Affiliations:** Department of Urology, Peking Union Medical College Hospital, Chinese Academy of Medical Sciences, Beijing, China; UCSF / VA Medical Center, UNITED STATES

## Abstract

**Objective:**

To evaluate the clinical efficacy of sorafenib as preoperative neoadjuvant therapy in patients with high risk renal cell carcinoma (RCC).

**Materials and Methods:**

Clinical data of 18 patients with high risk RCC who received surgery done successfully after preoperative neoadjuvant therapy with sorafenib in Peking Union Medical College Hospital (PUMCH) from April 2007 to October 2013 have been reviewed and analyzed in this study.

**Results:**

Among the 18 patients there were 13 male and 5 female, with a median age of 54.6 years. The objective response rate (ORR) of the operation on the selected patients is very high (94.4%), including 4 cases (22.2%) of partial response (PR) and 13 cases (72.2%) of stable disease (SD). After preoperative sorafenib treatment, the average tumor size of the 18 patients decreased from 7.8 cm (ranging from 3.6 to 19.2 cm) to 6.2 cm (ranging from 2.4 to 16.8 cm), and the median value of average tumor CT value decreased from 61HU to 52 HU. Among the 5 patients who had IVC tumor thrombi, the grades of tumor thrombi in 2 patients who were grade II before sorafenib treatment became grade I and grade 0 respectively, 2 patients of grade III both became grade II.

**Conclusion:**

Preoperative neoadjuvant therapy with sorafenib for high risk RCC patients can significantly decrease primary tumor volume as well as tumor thrombus, which could help the nephron-sparing surgery (NSS) or radical nephrectomy to be done successfully.

## Introduction

The incidence of renal cell carcinoma (RCC) is increasing worldwide over recent years[1–3]. With the advancement of medical imaging technology and the popularization of individual physical examination, most patients are diagnosed with RCC at localized stage. However, there are still about 20–30% of patients who are found having distal metastasis or advanced RCC at the time of diagnosis, which make them inadequate to undergo the operation[4]. Since RCC is highly resistant to chemotherapy and radiotherapy, while its response to cytokine therapy is less than 20%[5], new treatment strategies for advanced RCC are urgently needed. In recent years, the introduction of new agents targeting tumor angiogenesis and intracellular pathways has dramatically changed the therapeutic approach for RCC. Through preoperative neoadjuvant therapy with targeted drugs unresectable RCCs can be downsized so as to be surgically removed, which greatly increases the survival rate of RCC patients.

Sorafenib, one of these new targeted drugs, is a multi-targeting tyrosine kinase inhibitor against VEGF receptors, platelet-derived growth factor receptors, Fms-like tyrosine kinase 3, RET, KIT, and the RAF serine/threonine kinases[6]. Phase II and Phase III trials have demenstrated the significant therapeutic effect of sorafenib in RCC[7,8]. Sorafenib was approved by the State Food and Drug Administration in the People’s Republic of China as first-line/second-line treatment for advanced RCC in 2007. As the first targeted drug entering China’s market, sorafenib has been widely accepted because the well tolerance and significant efficacy in Chinese population[9,10]. In this study, clinical data of 18 high risk RCC patients who underwnt surgery successfully after preoperative neoadjuvant therapy with sorafenib in Peking Union Medical College Hospital (PUMCH) from April 2007 to October 2013 have been reviewed to study the efficacy and safety of sorafenib in RCC patients.

## Material and Methods

### Patient selection

A total of 173 RCC patients was treated with sorafenib in Peking Union Medical College Hospital (PUMCH) from April 2007 to October 2013. Among them 136 patients received postoperative treatment with sorafenib were excluded from this study. For the left 37 RCC patients who received preoperative neoadjuvant therapy with sorafenib, the inclusion criteria was patients who received surgery done successfully. Finally clinical data of 18 RCC patients who received surgery done successfully after preoperative neoadjuvant therapy with sorafenib were selected and reviewed in this work. The study was approved by the Ethics Committee of Peking Union Medical College Hospital. Written informed consent was obtained from every subject.

### Clinical data analysis

Clinical data of the patients were divided into 4 sections: 1. Demographic characteristics including age, sex, tumor size, tumor stage, preoperative aspiration analysis, and the dosage, duration, adverse events of targeted drug therapy, as well as preoperative drug withdraw time; 2. Change of tumor imaging study before and after treatment, by comparing chest, abdomen and pelvic CT scan with contrast in all patients before and after treatment. Major indicators include maximum tumor diameter, length of inferior vena cava thrombus, tumor CT value in enhanced scan, and distal metastasis; 3. Choice of surgery, time of operation, intra-operative blood loss, surgical complications and post-operative recovery; 4. Postoperative treatment and follow-up.

## Results

### Demographics characteristics

Among the 18 patients there were 13 male and 5 female, with a median age of 54.6 years (age ranging from 38 to 76 years). Tumor stages were divided according to the 2009 AJCC criteria, with 3 cases in stage I, 2 cases in stage II, 5 cases in stage III and 8 cases in stage IV. 11 cases had local advanced RCC, and 7 cases had distal metastasis (5 cases with lung metastasis, 1 case with lung and bone metastasis, 1 case with lymph node metastasis). Based on the pathology of renal cell cancers diagnosed by preoperative tumor aspiration 15 clear cell RCC, 1 chromophobe RCC and 2 unclassified RCC were determined. Inferior vena cava (IVC) tumor thrombi formation was detected in 5 patients, which could be divided as 2 cases of grade II, 2 cases of grade III and 1 cases of grade IV according to Mayo Clinic staging system.

The dose of sorafenib for preoperative targeted therapy was 400 mg orally twice daily, with an average treatment duration of 96 days (ranging from 30–278 days). All adverse events were within grade 3, including extremity skin reaction (16 cases, 88.9%), blood pressure elevation (11 cases, 61.2%), diarrhea (10 cases, 55.6%), alopecia (9 cases, 50%), rashes (8 cases, 44.5%), oral mucositis (8 cases, 44.5%), anemia (3 cases, 16.7%), transaminase elevation (1 case, 5.6%). There were no recorded adverse events over grade 4.

After receiving sorafenib neoadjuvant therapy for 30 days, 1 patient dropped out from drug therapy and underwent surgery because of drug intolerance. The dose of sorafenib had to be decreased to 600 mg/day in 2 patients in consequence of adverse events. Drugs were withdrawn 7–30 days before the surgery, with an average time of 12 days.

The demographics characteristics of the patients were listed in Table 1.

**Table 1:** Patient Characteristics.

**Characteristics**	**No.(%)**	**Range**
Median age (year)	54.6	38–76
Sex		
Male	13(72.2%)	
Female	5(27.8%)	
Stage		
I	3(16.7%)	
II	2(11.1%)	
III	5(27.8%)	
IV	8(44.4%)	
Local Advanced	11(61.1%)	
Metastatic RCC	7(38.9%)	
Pathology		
Clear Cell	15(83.3%)	
Chromphobe	1(5.6%)	
Other(no classification)	2(11.1%)	
Median duration of therapy (day)	96	30–278
Median time off treatment prior to surgery (day)	12	7–30

### Efficacy of preoperative neoadjuvant therapy with sorafenib

**Figure 1:**
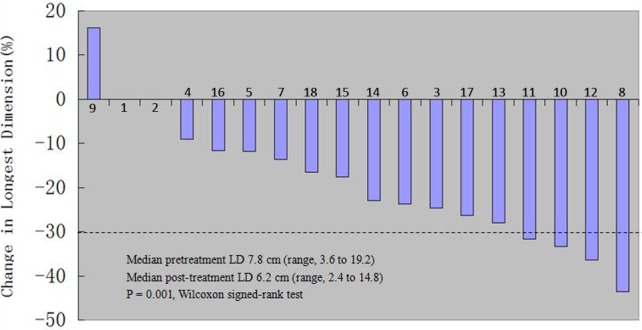
Change of the tumor size in the 18 patients.

**Figure 2:**
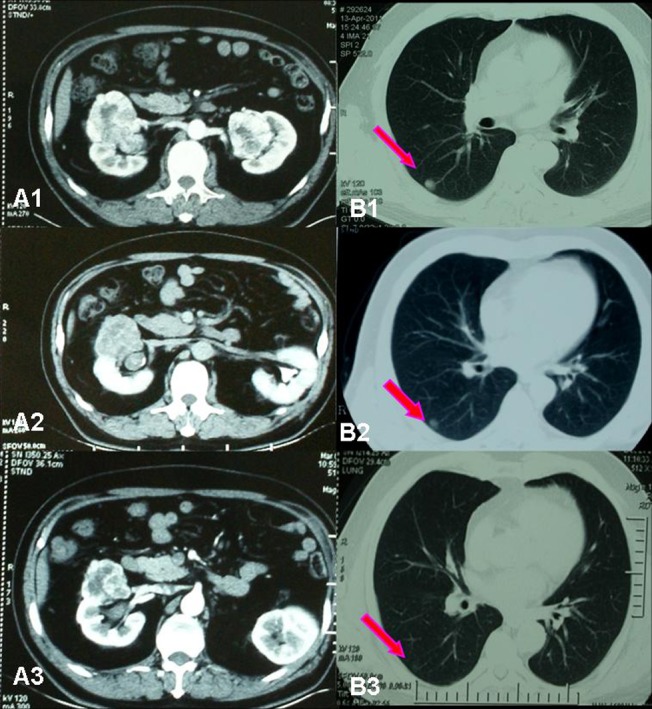
Imaging data of No. 17 patient who had bilateral RCC with lung metastasis. (A1) Before neoadjuvant therapy, maximum tumor diameter in right kidney was 7 cm, maximum tumor diameter in left kidney was 4 cm. (B1) Right lung metastasis diameter was 1.5 cm. (A2) After left partial nephrectomy and successive 90 days of neoadjuvant therapy, maximum tumor diameter in right kidney was reduced to 5.2cm. (B2) Right lung metastasis diameter was 1.0 cm. (A3) After 150 days of neoadjuvant therapy, maximum tumor diameter in right kidney was 4.6 cm. (B1) Right lung metastasis diameter was 0.5 cm.

Tumor response was assessed using RECIST criteria[11]. Because the patients recruited in this study were those who had significant improvement and were suitable for operative treatment, the objective response rate (ORR) of the operation on the selected patients is very high (94.4%), including 0 cases (0%) of complete response (CR), 4 cases (22.2%) of partial response (PR), 13 cases (72.2%) of stable disease (SD) (Table 2). The average tumor size (the longest dimension) of the 18 patients before sorafenib treatment was 7.8 cm (ranging from 3.6 to 19.2 cm), while the average tumor size after sorafenib treatment was 6.2 cm (ranging from 2.4 to 16.8 cm). Notably, there is 1 patient (5.6%) who had tumor progression after preoperative sorafenib treatment (Patient No. 9 in Table 3), 2 patients (11.1%) who had no change in tumor size (Patient No. 1 & 2 in Table 3). All other 15 patients (83.3%) had decreased tumor size at different degree (Fig. 1). In 1 case of bilateral RCC patient who had lung metastasis, after receiving oral sorafenib neoadjuvant therapy for 150 days following left partial nephrectomy, the primary tumor in the right kidney and the lung metastasis were both decreased gradually, and right partial nephrectomy was performed succesfully (Patient No. 17, Fig. 2).

**Table 2:** RECIST assessment in the 18 RCC patients.

	**n**	**%**
Complete response (CR)	0	0
Partial response (PR)	4	22.2
Stable disease (SD)	13	72.2
Progressive disease (PD)	1	5.6

RECIST, response evaluation criteria in solid tumors.

**Table 3:** Treatment details of each patient.

**Patient number**	**Treatment time (day)**	**Cancer size (diameter/cm)**		**Average tumor CT value (Hu)**		**Tumor Stage**		**IVC tumor thrombi grades**		**Surgery**	**Pathology**	**Time of follow-up (day)**	**PFS (day)**
		**Before**	**After**	**Before**	**After**	**Before**	**After**	**Before**	**After**				
1	30	6.5	6.5	55	52	IV	IV	-	-	radical nephrectomy	clear cell carcinoma	196	180
2	60	5.6	5.6	48	36	I	I	-	-	radical nephrectomy	clear cell carcinoma	164	164
3	68	5.7	4.3	95	54	IV	IV	-	-	radical nephrectomy	clear cell carcinoma	287	126
4	69	6.7	6.1	38	38	III	III	-	-	radical nephrectomy	no classfication	118	108
5	70	5.1	4.5	33	42	I	I	-	-	radical nephrectomy	clear cell carcinoma	256	246
6	72	3.8	2.9	69	55	III	III	II	0	rad. nep. + IVC. thr.	clear cell carcinoma	48	48
7	77	5.9	5.1	43	29	IV	IV	-	-	radical nephrectomy	clear cell carcinoma	214	214
8	80	7.8	4.4	88	72	II	I	-	-	radical nephrectomy	clear cell carcinoma	32	32
9	83	6.2	7.2	68	56	IV	IV	-	-	radical nephrectomy	no classfication	76	76
10	86	3.6	2.4	32	26	I	I	-	-	partial nephrectomy	chromphobe	145	128
11	86	15.2	10.4	73	59	IV	IV	-	-	radical nephrectomy	clear cell carcinoma	126	126
12	90	11.3	7.2	52	47	III	III	III	II	rad. nep. + IVC. thr.	clear cell carcinoma	83	83
13	92	7.5	5.4	54	51	III	III	III	II	rad. nep. + IVC. thr.	clear cell carcinoma	228	228
14	94	19.2	14.8	72	59	IV	IV	-	-	radical nephrectomy	clear cell carcinoma	65	65
15	96	5.1	4.2	56	45	II	II	-	-	radical nephrectomy	clear cell carcinoma	362	362
16	148	7.7	6.8	49	43	IV	IV	IV	IV	rad. nep. + IVC. thr.	clear cell carcinoma	740	603
17	150	7.0	5.2	116	112	IV	IV	-	-	partial nephrectomy	clear cell carcinoma	91	91
18	278	10.3	8.6	57	52	III	III	II	I	rad. nep. + IVC. thr.	clear cell carcinoma	48	48

Before, before drug treament; After, after drug treament; rad. nep. + IVC. thr., radical nephrectomy+IVC thrombothromboembolectomy; PFS, progression free survival.

**Figure 3:**
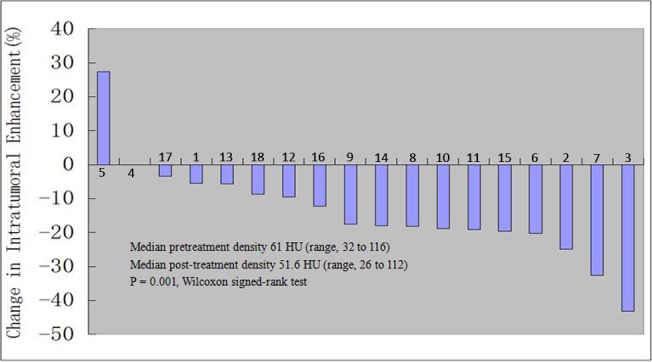
Change of the average tumor CT value in the 18 patients.

**Figure 4:**
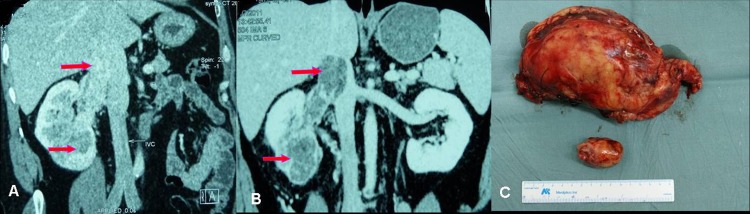
Imaging data of No. 11 patient who had left RCC with renal hilar lymph node metastasis. (A) Left kidney tumor and renal hilar lymph node metastasis before neo-adjuvant therapy. (B) After 86 days of neoadjuvant therapy, left kidney tumor downsized and renal hilar lymph node decreased, tumor partially necrotized. (C) Section of resected left kidney, showing partial tumor necrosis and fibrosis.

Before preoperative sorafenib treatment, the median value of average tumor CT value in enhancement phase detected by CT scan was 61HU (ranging from 32 to 116 HU). After sorafenib treatment, the median value of average tumor CT value was 52 HU (ranging from 26 to 112 HU) (Fig. 3). The decrease in tumor CT value might due to the tumor necrosis or fibrosis caused by sorafenib neoadjuvant therapy (Fig. 4).

**Figure 5:**
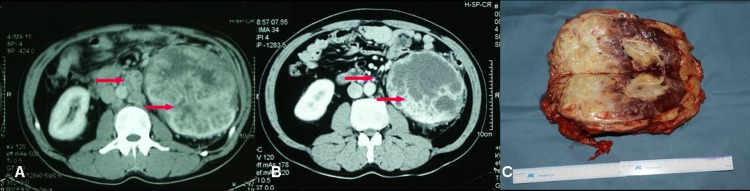
Imaging data of No. 13 patient who had right RCC with tumor thrombus. (A) Right RCC with IVC grade III tumor thrombus before neoadjuvant therapy. IVC was obliterated. (B) After 92 days of neoadjuvant therapy, IVC thrombus was down–graded to grade II, IVC was partially recanalized. (C) Resected right kidney and IVC tumor thrombus.

Among the 5 patients who had IVC tumor thrombi, the grades of tumor thrombi in 2 patients who were grade II befor sorafenib treatment became grade I and grade 0 respectively; 2 patients of grade III became grade II (Fig. 5), the last patient with grade IV tumor thrombi had no change in the grades, but the thrombus decreased significantly from the level of right atrium to above diaphragm.

### Surgical treatment after neoadjuvant therapy

**Figure 6:**
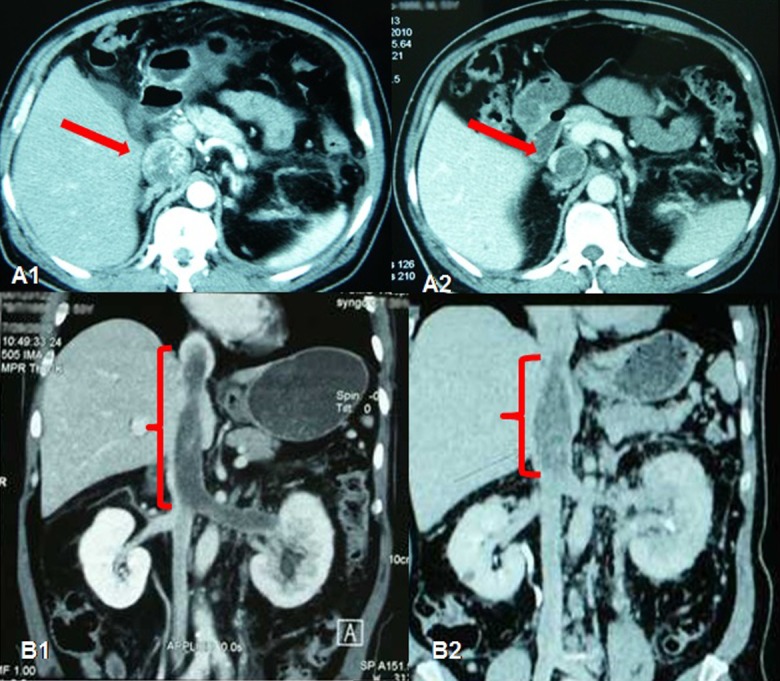
Imaging data of No. 16 patient with obstructed inferior vena cava (ICV). (A1) IVC tumor thrombus filled IVC before neo-adjuvant therapy. (A2) After 148 days of neo-adjuvant therapy, IVC thrombus was shortened and fibrotic. (B1) IVC thrombus reached right atrium before neo-adjuvant therapy. (B2) After 148 days of neoadjuvant therapy, IVC thrombus was shortened.

All the 18 patients received surgery after serafenib neoadjuvant therapy, with open surgery in 13 cases(72.2%) and laparoscopy in 5 cases (27.8%). 2 patients (11.1%) underwnt partial nephrectomy after diminution of tumor size, with 1 open surgery and 1 laparoscopy. 11 patients (61.1%) received radical nephrectomy, with laparoscopy in 3 cases. 4 patients performed radical nephrectomy and IVC thromboembolectomy, with 3D laparoscopy in 1case. A patient who had left RCC and IVC thrombus of grade IV received radical nephrectomy combined open thoracic IVC thromboembolectomy under extracorporeal circulation (Fig. 6).

The average time of operation was 156 min, ranging from 85min to 370min. The average amount of intraoperative blood loss was 380 ml, with a range of 150 to 4000 ml. There was one patient who received radical nephrectomy and IVC thromboembolectomy had postoperative hemorrhage 5 hours after the surgery, and reoperation was afforded to stop bleeding. No other surgical complications were observed. Surgical wounds healed well and sutures were removed as expected in all cases. Time of postoperative hospital stay varied from 5 days to 28 days, with an average of 11 days.

### Postoperative treatment and follow-ups

**Figure 7:**
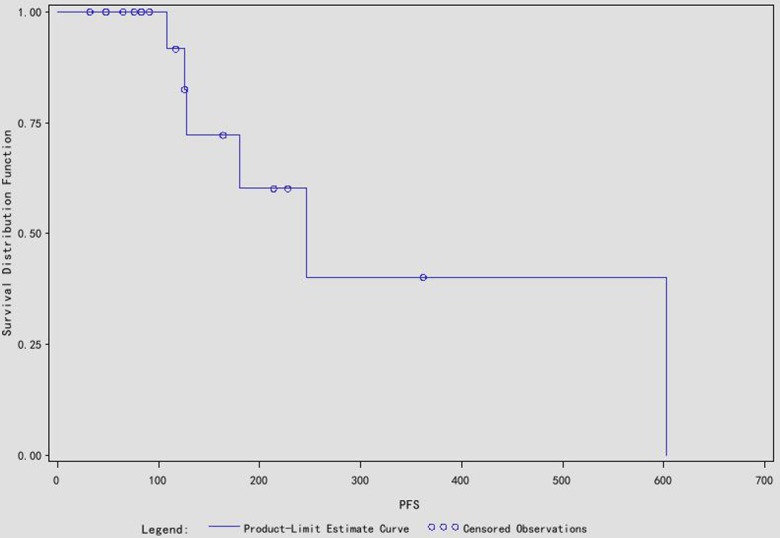
The survival curve of the 18 cases.

After the operation 7 patients who had distal metastasis at the time of diagnosis received targeted drug therapy again within 2–4 weeks. Among them 2 patients had distal metastasis progression at 4 month and 9 month and the treatment drug was replaced with everolimus. In the 11 local advanced RCC cases, 5 patients continued sorafenib treatment after the surgery and 1 of them who had localized RCC recurrence was treated with everolimus alternatively; 3 patients had distal metastasis and were retreated with sorafenib, 3 patients dropped out from drug treatment for observation. The follow-up of the patients was 186 days (ranging from 32 to 740 days), and all the 18 patients survived over follow-up period (Fig. 7). The median progression free survival (PFS) was 246 days, and the mean PFS was 346.6 days.

## Discussion

So far, surgery still represents the best curative option for patients with renal cell carcinoma (RCC). Though most RCCs can be diagnosed at early stage with the popularization of individual physical examination, there are still a significant amount of patients having distal metastasis or advanced RCC at the time of diagnosis, which make it unsuitable for them to undergo the surgery treatment. Besides, there are still some patients called high risk RCC patients who are at high risks to perform operation. Patients with the following situations can be diagnosed as high risk RCC: 1, Patient with long length inferior vena cava thrombus and the Mayo clinic grade≥II; 2, Patients with large tumor (the diameter>7cm); 3, Patient who has double kidney tumors or multiple tumors and is undergoing NSS; 4, Patient who has tumors in the anatomical or functional solitary kidney and is unsuitable to undergo NSS; 5, Patient who has widespread metastatic renal cell carcinoma and temporarily resection of the primary tumor has no effect.

In recent years, considerable progress has been made in the treatment of patients with renal cell carcinoma, with new targeted drugs and systemic strategies revolutionising the management of this disease. Many targeted drugs have been introduced into RCC treatment over recent years, which greatly impact the therapeutic approach to metastatic RCCs[12,13]. Preoperative neoadjuvant therapy and postoperative adjuvant therapy with new targeted drugs have been studied in many medical centers [14,15]. Notably, the safety and efficacy of the preoperative neoadjuvant therapy have been demonstrated and recognized by many clinical practices and provoked great expectation[16,17].

What’s the theoretical foundation of neoadjuvant therapy with targeted drug? With the accumulation of clinical experience, it is realized that the main function of targeted drugs is to suppress and stabilize the tumors, so it is impossible to achieve complete cure with targeted drugs alone. Surgery still represents the only curative and irreplaceable method for RCC patients. However, targeted drug therapy can decrease the tumor size and lower the tumor grades, thereby make it possible to surgically resect the tumor, at the same time reducing the risks and difficulties in the surgery. Thus, the combination of targeted drug therapy and surgery in the treatment of high risk RCC patients is worthy of studying and exploration, especially preoperative neoadjuvant therapy with targeted drugs.

As one of the new targeted drugs, sorafenib is a multi-targeting tyrosine kinase inhibitor against VEGF receptors such as VEGFR-2 and VEGFR-3, platelet-derived growth factor receptors, Fms-like tyrosine kinase 3, RET, KIT, and the RAF serine/threonine kinases[6]. Sorafenib can block the activation of Ras/Raf Kinase singling induced by Ras mutation so as to inhibit MEK/MAPK/ERK signaling and induce apoptosis. Phase III trials have demenstrated the significant therapeutic effect of sorafenib by increasing median progression free survival in metastatic RCC patients[7,8]. Sorafenib was approved as the first targeted drug in the treatment of advanced renal cancer by the U.S. Food and Drug Administration (FDA) in December 2005. Sorafenib was introduced into China’s market and approved by the State Food and Drug Administration in the People’s Republic of China in 2007. Since then the high efficacy and safety of sorafenib in the treatment of RCC among Chinese population have been demonstrated by multiple clinical studies [9,18].

Our study here provided a solid basis for the effectiveness and advocacy of preoperative neoadjuvant therapy in high risk RCC patients. The neoadjuvant therapy with targeted drugs are recommended under the following conditions: 1, long length inferior vena cava thrombus with Mayo clinic grade≥II; 2, large tumor (the diameter>7cm); 3, double kidney tumors or multiple tumors and NSS is taken into consideration; 4, tumors in the anatomical or functional solitary kidney and is unsuitable to undergo NSS; 5, widespread metastatic renal cell carcinoma and temporarily resection of the primary tumor has no effect; 6, none above conditions, but the patient is severely ill and the cardiac and pulmonary function need to be improved. For patients with above conditions, preoperative neoadjuvant therapy with targeted drugs is the best therapeutic approach to prepare patients for surgery. There are some patients who prefer continually drug therapy than receiving further surgery because of the obvious effect. However there are still some patients showing no obvious improvement because of drug resistance during the neoadjuvant therapy.

Commonly it would be 3 months for preoperative drug neoadjuvant therapy before operation. The time of operation could be determined by the doctors if the tumor volume decreased to the appropriate size for surgery. However in our study the duration of neoadjuvant therapy was nterrupted by multiple unexpected factors in the process, including the efficacy of drug therapy, adverse events, personal willingness of patients, economical factors and so on. As a result, the actual duration of drug therapy varied from 30 days to 278 days. In the shortest duration case, surgery had to be brought forward for drug intolerance. In the longest case, the patient initially refused surgery and prefered continual drug therapy because of the excellent early response, however after 8 months of durg treatment the surgery had to be taken because of the tumor progression. Two important facts were learned through practice. first, according to intraoperative observation, adhesion between tumor and surrounding tissue increased with the time of neoadjuvant therapy extended. Second, The decreasing rate of tumor size in the late phase of the treatment was not as significant as in the early phase. Thus, we would suggest the optimal duration of neoadjuvant therapy should be around 2–4 months.

Theoretically speaking, since sorafenib has a relatively short half-life (25–48 hours), surgery can be performed 3 days after drug withdrawing. However, considering the optimal time for wound healing, surgery was performed 7 or more days after drug withdrawing. As a result, all 18 cases had uncomplicated wound healing. Shorter withdraw interval can be tested in future practice. While targeted drug therapy inhibits tumor angiogenesis, normal tissue blood supply can also be compromised, and the risk of collateral damage during surgical dissection is increased. Yet, all 18 cases showed no collateral tissue damage during surgery, which suggested the minor influence of targeted drug therapy on normal viscera or careful and delicate surgical manipulation might avoid collateral tissue damage.

CT imaging study showed tumor size decrease at various degree in 15 patients (83.3%). More notably, it was observed that tumor thrombi was shortened at various degrees in the 5 cases with IVC tumor thrombi which made the surgical treatment possible. CT scan with contrast can directly demonstrate tumor blood supply, which is closely related to tumor angiogenesis, thus CT value can indirectly reflect the suppression efficacy of targeted drugs on tumor angiogenesis [19]. Most tumor downsizing cases were also accompanied by CT value changes, possibly due to tissue liquidation, necrosis and fibrosis after decrease in tumor blood supply [20]. On the other hand, CT value increase might indicate tumor progression.

In post-surgical follow-ups, there were both distal metastatic cases and local advanced RCC cases showed further progression. For these cases treatment with a mTOR inhibitor, everolimus was conduvted. Due to the limited case numbers and follow-up period, long term efficacy of neoadjuvant therapy still needs further investigation.

## Conclusion

Preoperative neoadjuvant therapy with sorafenib can decrease the primary tumor size, reduce tumor blood supply, induce tumor necrosis and fibrosis in high risk RCC patients, therefore increase safety of surgery. In patients with IVC tumor thrombi, neoadjuvant therapy with sorafenib can shorten the length of tumor thrombi and induce fibrosis which benefit the further thromboembolectomy. The preoperative neoadjuvant therapy with sorafenib combined surgery is of great effectiveness in high risk RCCpatients among Chinese population and worth popularization.
